# Supplementary material for: A transformation from temporal to ensemble coding in a model of piriform cortex
Source: eLife. 2018 Mar 29;7:e34831. doi: 10.7554/eLife.34831 (PMC5902166; doi:10.7554/eLife.34831)
Supplement: Source code 1. — This C code is used in an environment that can execute consecutive single steps and plot the results (e.g. xcode). [file elife-34831-code1.rtf]

/******* NOTES *******/// Notes// This code was written by Merav Stern and Larry Abbott and was designed by Merav Stern, Larry F Abbott and Kevin Franks// Released 22 March 2018// This code is the just the core of the program, it doesn't run by itself but rather requires an environment that can execute consecutive single steps and plot the results./******* DEFINES *******/#define tau				0.015#define tauEx			0.02 #define tauIn			0.01#define tauMit          0.005#define tauMdecay       0.05#define tRef			0.001 #define VTh				-50.0#define	VMin			-75.0#define VReset			-65.0#define nCell			12450#define nExCell			10000#define sqExCell		100#define nInCellFB		1225#define sqInCellFB		35#define nInCellFF		1225#define sqInCellFF		35#define nG				900#define	nM				25#define sqGlom			30#define nLOT			50#define nEx				1000#define	nIn				50#define lInConnect      2#define maxConnect		2000#define	tSniff			0.3#define tInhale			0.2#define tExhale			0.1#define nMaxMax			10#define rMmax           100#define nOdor			25#define nMaxSpike		1000#define pLOT			0.1#define maxSpikeCount	20.0/******* GLOBALS *******/double 	*V, *tSLS, *gEx,*gMit, *gInFF, *gInFB, *iBias, iMag, iSig;double	dGExEx, dGInFF, dGInFB, dGLEx, dGLIn, dGExIn, expV, expFacEx, expFacMit, expFacIn, LotBackRate;double	**tLOT, c, *randSig;int		**goesTo, *nConnect, nT, iOdor, *nS, **mList, tIn, tSn, **mTo, nM2P, iT,nLotBackSpikes;void	NewOdor(void);/******* SET GLOBALS *******/void SetGlobals(void){    int		i, j, k, K, tempCell, tc, p;    double     iPlaceVrt, iPlaceHrz, jPlaceVrt, jPlaceHrz;        V = Vector(nCell);    tSLS = Vector(nCell);    gEx	= Vector(nCell);    gMit	= Vector(nCell);    gInFF	= Vector(nCell);    gInFB	= Vector(nCell);    iBias = Vector(nCell);    randSig = Vector(nExCell);    goesTo = IMatrix(nCell, maxConnect);    nConnect = IVector(nCell);        for (i=0; i<nCell; i++)        V[i] = VReset;        //reccurent excitatory    for (i=0; i<nExCell+nInCellFB; i++)    {        k = 0;        K = 0;        j = 0;        while (nEx>k)        {            if (j==i)                j++;            p = (double) (nEx-k)/(double) (nExCell-K-1);            if (Randx()<p)            {                goesTo[j][nConnect[j]] = i;                if (nConnect[j]<maxConnect-1)                    nConnect[j]++;                else                    overFlow = true;                k++;            }            j++;            K++;        }    }        //global feedforward inhibition    for (i=0; i<(nExCell+nInCellFF); i++)    {        if (i<nExCell) {            tempCell = i;        } else {            tempCell = i+nInCellFB;        }        k = 0;        K = 0;        j = nExCell+nInCellFB;        while (nIn>k)        {            if (j==i)                j++;            p = (double) (nIn-k)/(double) (nInCellFF-K-1);            if (Randx()<p)            {                goesTo[j][nConnect[j]] = tempCell;                if (nConnect[j]<maxConnect-1)                    nConnect[j]++;                else                    overFlow = true;                k++;            }            j++;            K++;        }    }        //local feedback inhibition    tc = 0;    for (i=0; i<nInCellFB; i++)    {        iPlaceVrt = floor(i/sqInCellFB)*sqExCell;        iPlaceHrz = fmod(i,sqInCellFB)*sqExCell;        for (j=0; j<nExCell+nInCellFB; j++) {            if (j==i) {            }            else {                if (j<nExCell) {                    jPlaceVrt = floor(j/sqExCell)*sqInCellFB;                    jPlaceHrz = fmod(j,sqExCell)*sqInCellFB;                } else {                    jPlaceVrt = floor((j-nExCell)/sqInCellFB)*sqExCell;                    jPlaceHrz = fmod(j-nExCell,sqInCellFB)*sqExCell;                }                if ((iPlaceVrt-jPlaceVrt)*(iPlaceVrt-jPlaceVrt)+(iPlaceHrz-jPlaceHrz)*(iPlaceHrz-jPlaceHrz)<(lInConnect*sqExCell)*(lInConnect*sqExCell)) {                    goesTo[i+nExCell][nConnect[i+nExCell]] = j;                    nConnect[i+nExCell]++;                    if (j>nExCell) {                        tc++;                    }                }            }        }    }        for (i=0; i<nExCell; i++)        randSig[i] = RandGauss();        nM2P = ceil((double)((nExCell+nInCellFF)*nLOT)/(double)(nG*nM));    nT = floor(duration/dt) + 1;    tIn = floor(tInhale/dt) + 1;    tSn = floor(tSniff/dt) + 1;    tLOT = Matrix(nOdor, nG);    mList = IMatrix(tSn, nMaxSpike);    nS = IVector(tSn);    mTo = IMatrix(nM*nG, nM2P);        for (i=0; i<nM*nG; i++)        for (j=0; j<nM2P; j++){            tempCell = floor((nExCell+nInCellFF)*Randx());            if (tempCell<nExCell) {                mTo[i][j] = tempCell;            } else {                mTo[i][j] = tempCell+nInCellFB;            }        }        for (i=0; i<nOdor; i++)        for(j=0; j<nG; j++)            tLOT[i][j] = tInhale*Randx()/pLOT;}/******* PARAMETERS *******/void SetParameterValues(void){    int		i;        c = 1.00;    dGExIn = 1.0;    dGExEx = 0.25;    dGLEx = 10.0;    dGLIn = 10.0;    dGInFF = 10.0;    dGInFB = 10.0;    iMag = -0.5;    iSig = 2.0;    for (i=0; i<nExCell; i++)        iBias[i] = iMag + iSig*randSig[i];    for (i=nExCell; i<nCell; i++)        iBias[i] = 0.0;    LotBackRate = 1.5; // or 2.0    nLotBackSpikes = floor(LotBackRate*tSniff*nM*nG);    iOdor = 0;        expV = exp(-dt/tau);    expFacEx = exp(-dt/tauEx);    expFacIn = exp(-dt/tauIn);    expFacMit = exp(-dt/tauMit);}/******* BEFORE RUN *******/void BeforeRun(void){    NewOdor();    iT = tSn-1;}/******* RUN ONE STEP *******/void RunOneStep(void){    double	VInf;    int		i, j, k, m, iP, iLOT, iCell;        if ((iT>=0))    {        for (iP=0; iP<nS[iT]; iP++)        {            m = mList[iT][iP];            for (iLOT=0; iLOT<nM2P; iLOT++)            {                iCell = mTo[m][iLOT];                if (iCell<nExCell)                    gMit[iCell] += dGLEx;                else if (iCell>nExCell+nInCellFB)                    gMit[iCell] += dGLIn;            }        }        iT--;    }    for(i=0; i<nCell; i++)    {        if (gEx[i]>1.0e-10)            gEx[i] *= expFacEx;        if (gMit[i]>1.0e-10)            gMit[i] *= expFacMit;        if (gInFF[i]>1.0e-10)            gInFF[i] *= expFacIn;        if (gInFB[i]>1.0e-10)            gInFB[i] *= expFacIn;        if (tSLS[i]>0.0)        {            tSLS[i] -= dt;            V[i] = VReset;        }        else        {            VInf = VReset + gMit[i] + gEx[i] - gInFF[i] -gInFB[i]+ iBias[i];            V[i] = VInf + (V[i] - VInf)*expV;            if (V[i]<VMin)                V[i] = VMin;        }    }        for(i=0; i<nCell; i++)    {        if (V[i]>VTh)        {            V[i] = VReset;            tSLS[i] = tRef;            if (i<nExCell)            {                    for (k=0; k<nConnect[i]; k++)                    {                        j = goesTo[i][k];                        if (j<nExCell)                        {                            gEx[j] += dGExEx;                        } else {                            gEx[j] += dGExIn;                                                    }                    }            }            else if ((i>=nExCell)&&(i<(nExCell+nInCellFB)))            {                for (k=0; k<nConnect[i]; k++)                    gInFB[goesTo[i][k]] += dGInFB;            } else if (i>=(nExCell+nInCellFB))            {                    for (k=0; k<nConnect[i]; k++)                        gInFF[goesTo[i][k]] += dGInFF;            }        }    }}/******************************************************************** ADDITIONAL PROCEDURES *********************************************************************/void NewOdor(void){    int		tSpike, iG, nSpike, iTindx, iP, inM, iTM;    double	tForSpike, rMcur;    Boolean	overFlow = false;        for (iTindx=0; iTindx<tSn; iTindx++)    {        nS[iTindx] = 0;    }    for (iTindx=0; iTindx<tIn; iTindx++)    {
